# Dynamics of *Bacillus thuringiensis* var. *israelensis* and *Lysinibacillus sphaericus* Spores in Urban Catch Basins after Simultaneous Application against Mosquito Larvae

**DOI:** 10.1371/journal.pone.0055658

**Published:** 2013-02-04

**Authors:** Valeria Guidi, Angelika Lehner, Peter Lüthy, Mauro Tonolla

**Affiliations:** 1 Microbial Ecology Group, Microbiology Unit, Plant Biology Department, University of Geneva, Genève, Switzerland; 2 Institute of Microbiology, Bellinzona, Switzerland; 3 Institute for Food Safety and Hygiene, Vetsuisse Faculty, University of Zurich, Zürich, Switzerland; 4 Institute of Microbiology, ETH Zürich, Zürich, Switzerland; International Atomic Energy Agency, Austria

## Abstract

*Bacillus thuringiensis* var. *israelensis* (Bti) and *Lysinibacillus sphaericus* (Lsph) are extensively used in mosquito control programs. These biocides are the active ingredients of a commercial larvicide. Quantitative data on the fate of both Bti and Lsph applied together for the control of mosquitoes in urban drainage structures such as catch basins are lacking. We evaluated the dynamics and persistence of Bti and Lsph spores released through their concomitant application in urban catch basins in southern Switzerland. Detection and quantification of spores over time in water and sludge samples from catch basins were carried out using quantitative real-time PCR targeting both *cry4A* and *cry4B* toxin genes for Bti and the *binA* gene for Lsph. After treatment, Bti and Lsph spores attained concentrations of 3.76 (±0.08) and 4.13 (±0.09) log ml^−1^ in water, then decreased progressively over time, reaching baseline values. For both Bti and Lsph, spore levels in the order of 10^5^ g^−1^ were observed in the bottom sludge two days after the treatment and remained constant for the whole test period (275 days). Indigenous Lsph strains were isolated from previously untreated catch basins. A selection of those was genotyped using pulsed field gel electrophoresis of *Sma*I-digested chromosomal DNA, revealing that a subset of isolates were members of the clonal population of strain 2362. No safety issues related to the use of this biopesticide in the environment have been observed during this study, because no significant increase in the number of spores was seen during the long observation period. The isolation of native *Lysinibacillus sphaericus* strains belonging to the same clonal population as strain 2362 from catch basins never treated with Lsph-based products indicates that the use of a combination of Bti and Lsph for the control of mosquitoes does not introduce non-indigenous microorganisms in this area.

## Introduction

The biolarvicides *Bacillus thuringiensis* var. *israelensis* de Barjac (Bti) and *Lysinibacillus sphaericus* Meyer and Neide (Lsph), formerly known as *Bacillus sphaericus* [1], are widely used for the control of a variety of mosquito species and, with regards to Bti, for black flies [2], [3]. *Lysinibacillus sphaericus sensu latu* is a very heterogeneous species, with 5 main DNA homology groups [4] with extremely variable intergroup sequence homologies (20 to 60%). Group II is formed by subgroups IIA and IIB. All entomopathogenic strains belong to the homology group IIA and can be divided into high-toxicity and low-toxicity types [5]. The larvicidal activity of highly pathogenic Lsph is primarily the consequence of the action of the binary toxins (BinA and BinB proteins) produced during the sporulation phase and enclosed within the exosporium [2], [6]. Recently, a two-component toxin (Cry48Aa-Cry49Aa binary toxin) was characterized in some Lsph strains able to overcome resistance to Bin toxins [7], [8]. In addition to crystal toxins, low-toxicity as well as some high-toxicity entomopathogenic Lsph strains produce mosquitocidal proteins (Mtx proteins) during the vegetative growth [2], [3]. *Bacillus thuringiensis* var. *israelensis* possesses a more complex arsenal of mosquitocidal toxins (Cry4Aa, Cry4Ba, Cry10Aa, Cry11Aa, Cyt1Aa, and Cyt2Ba, also referred as delta-endotoxins), encoded by genes located on the large pBtoxis plasmid [9] and enclosed within the parasporal crystal. The different mode of action of Cry and Cyt toxins and their synergistic interaction prevent the selection of resistance to Bti in the target organism [10], [11]. Bti is very effective and kills mosquito larvae within a few hours from the ingestion of the protoxins. Nevertheless, its short-term residual activity makes the use of Bti unsuitable for the control of multivoltine species or continuously breeding mosquitoes [12], [13], especially in polluted water or in liquids rich in organic material [14]. Lsph-based larvicides, in contrast to Bti, have a narrow host spectrum, with *Culex* spp. being the most susceptible mosquitoes. Species belonging to the genera *Anopheles*, *Aedes*, *Ochlerotatus*, and *Mansonia* are in general less sensitive [3], [15]. Lsph strains, however, have the advantage to persist and recycle in polluted water and to proliferate in cadavers of mosquitoes [3], even if the development of resistance against Lsph has been reported [16], [17], [18], [19], [20].

A biological mosquito larvicide (BML; VectoMax™ CG, Valent BioSciences Corp., Libertyville, IL, USA) containing living spores and crystals of both Bti and Lsph as active ingredients is available on the market. This product combines the properties of both microbial larvicides, providing quick kill of mosquito larvae and long-term residual control. Combinations of Bti and Lsph spores/toxins suspensions were proven to enhance the efficacy against the target species as a consequence of the synergic action between the toxins and to expand the host-range, at the same time reducing the pressure for the development of resistance against the Lsph component [21]. BML has been recently tested in field studies for the control of mosquitoes in catch basins [22] and in seasonal wetlands [23]. BML is thus a good candidate for the control of mosquitoes in polluted breeding sites with a continuous hatching and larval development, such as catch basins.

Persistence and multiplication are key components in the evaluation of the environmental safety of a bacterial pesticide. BML is a biological agent based on natural, genetically non-modified microorganisms that are deliberately introduced into the environment at high concentrations. Several studies have assessed the environmental persistence of *Bacillus* spores following applications [24], [25], [26], [27], [28], [29], [30], [31]. However, quantitative data on the fate of both Bti and Lsph applied together for the control of mosquitoes in urban drainage structures such as catch basins are lacking. Therefore, it is of importance to investigate the fate of Bti and Lsph following BML release into urban aquatic environments.

The goal of this study was to investigate the long-term dynamic and persistence of Bti and Lsph spores following BML application to urban catch basins. A real-time PCR methodology was developed to follow quantitatively spores in water and sludge samples under natural conditions.

## Materials and Methods

### Ethics Statement

The study area is located on public land. Permit for the described field study was obtained from the Swiss Federal Office of Health, Bern (Permit number 449.0037-6/12.004914/857685/).

### Study design and collecting methods

Twenty catch basins in the municipality of Chiasso, Switzerland were chosen for this study. In the past, mosquito breeding sites of this area were subjected to Bti treatments with a commercial larvicide (VectoBac-G®, Valent Biosciences Corp., Libertyville, USA) throughout a field program aiming at controlling the invasive Asian tiger mosquito (*Aedes albopictus* Skuse). Seventeen catch basins (T1 to T17) were randomly selected and treated with the microbial larvicide BML (Bti serotype H14, strain AM65-52 and Lsph, serotype H5a5b, strain 2362; granules; 50 Lsph ITU mg^−1^). Three catch basins (UTC1, UTC2, and UTC3) served as untreated negative controls. Pre-treatment control sampling was carried out on 3 August 2011. Treatments were carried out on 8 August 2011 by applying 10 g product per catch basin, as recommended by the manufacturer. Post-treatment samples were collected at the same catch basins at days 2, 8, 21, 35, 49, 63 and 135 after BML application. A further sampling on 9 May 2012 (275 days post-treatment) was carried out in 4 treated catch basins (namely T2, T12, T14, and T16) and 1 UTC (UTC3). For each catch basin, one water and one sludge were sampled at each sampling using a standard dipper (Abbott Laboratories, Abbott Park, North Chicago, USA). One hundred ml of water were collected in a 125 ml screw cap pot and filtered through a 0.22 µm polycarbonate hydrophilic filter (Isopore™ membrane filters, Millipore AG, Zug, Switzerland) to concentrate bacteria immediately after the arrival at the laboratory. The filtered material was resuspended in 1 ml of distilled sterile water and stored at −20°C until processed. Sludge was collected into a 50 ml tube and kept at −20°C until processing.

### DNA extraction from sludge and water samples and quantitative real-time PCR

The concentration of Bti and Lsph in water was determined for all samples. Sludge samples were analyzed only for day 5 pre-treatment and days 2, 21, 49, 135 and 275 post-treatment.

Nucleic acid extractions were carried out according to Guidi et al. [32], starting from 400 µl of water and 0.25 g (dry weight) of sludge. The method was adapted for Lsph, reducing the spore germination step to 60 min.

Sample DNA was analyzed by real-time PCR targeting the *cry4Aa* and *cry4Ba* genes for Bti [30], and *binA* gene for Lsph. Primers amplifying a 102 bp sized fragment of the *binA* gene, as well as a TaqMan probe were designed using the Primer Express software ver. 3.0 (Applied Biosystems, Rotkreuz, Switzerland), and their sequences were checked for specificity in the GenBank database using BLAST (http://blast.ncbi.nlm.nih.gov/Blast.cgi). The BinA_Probe was labelled at the 5′ end with the fluorescent ATTO550 dye and at the 3′ end with the black hole quencher BHQ2 (Microsynth AG, Balgach, Switzerland). All primers and TaqMan probes used are listed in Table 1. The real-time PCR reactions were performed in a 7000 real-time PCR instrument (Applied Biosystems), with the following thermal cycling conditions: 2 min at 50°C, 10 min at 95°C followed by 40 cycles of 15 s at 95°C and 1 min at 60°C. Each reaction consisted of a 20 µl-solution containing 10 µl TaqMan® Environmental Master Mix 2.0 (Applied Biosystems), 0.9 µM of forward and reverse primers, 0.2 µM of probe, and 2 µl of template DNA. Absence of inhibition was checked by adding to each reaction a TaqMan® Exogenous Internal Positive Control-VIC™ Probe (Applied Biosystems). Water and sludge DNA extracts were analyzed in duplicates for both *cry4* and *binA* genes. Each amplification run included also a no template control sample and ten-fold standard dilutions for quantifications run in triplicate. Standard templates corresponded to DNA extracts from pure spore suspensions of known concentrations of Bti AM65-52 and Lsph 2362 [32]. Both strains were isolated from BML and spore suspensions prepared in liquid GYS medium [33] for 5 days at 30°C under shaking. Sporulation was checked by microscopy after Coomassie blue staining [34] and spores quantified by classical plate counting using blood agar plates (Columbia Agar with 5% sheep blood, Becton Dickinson AG, Basel, Switzerland). Real-time PCR data were analyzed with the 7000 System SDS software, version 1.2.3f2 (Applied Biosystems), with the automatic settings used for the baseline and threshold set at 0.4 Δ*Rn* for both *cry4* and *binA* genes.

**Table 1:** Primers and probe used for quantitative PCR in this study.

Organism	Primers or probe	Oligonucleotide sequence (5′-3′)	Oligonucleotide position* (gene amplified)	Reference
*B. thuringiensis israelensis*	Un4(d) (forward)	GCATATGATGTAGCGAAACAAGCC	589–612 (*cry4Aa*)	[51]
			2800–2823 (*cry4Ba*)	
	Cry4R (reverse)	ACCTGGAACATCTGACAACCAATC	454–477 (*cry4Aa*)	[30]
			2935–2958 (*cry4Ba*)	
	Cry4Probe	FAM-ACGACACTCGCTCAAATTCAGTACGCT-BHQ1	514–540 (*cry4Aa*)	[30]
			2872–2898 (*cry4Ba*)	
*L. sphaericus*	BinA_F (forward)	GAGAGGTCAAAAGAACTATGGCAACT	615–640 (*binA*)	This study
	BinA_R (reverse)	ATGGCAGCTCATGGGAATG	698–716 (*binA*)	This study
	BinA_Probe	ATTO550-CCTGAATCCGCAACAGATGTGAGAGCTC-BHQ2	662–689 (*binA*)	This study

* Starting from the first base of the sequence in the GenBank database (*cry4Aa*: accession number AL731825, region 92986 – 96528; *cry4Ba*: accession number AL731825, region 32597 – 36007; *binA*: accession number Y00378.1).

The size of the amplification products is 159 bp for the *cry4Aa* and *cry4Ba* genes and 102 bp for the *binA* gene.

The limit of detection (LOD) and the limit of quantification (LOQ) for both real-time PCR targeting the Bti *cry4* genes and the Lsph *binA* gene were determined by testing triplicate dilutions of standard samples on three separate assay runs, for a total of 9 replicates for each dilution. The detection limit was defined as the lowest number of spores in a sample required to obtain a positive result in 95% of samples analyzed [35]. The LOQ was defined as the lowest concentration tested with a relative standard deviation (RSD) below 25% [36].

### Isolation and characterization of *L. sphaericus*

Lsph was isolated from water and sludge samples collected before treatment that gave a positive amplification by real-time PCR. Isolates were further characterized by molecular analysis. BML 2362 and *L. sphaericus* ATCC14577^T^ (type strain) were used as reference strains.

DNA templates for molecular characterizations were prepared from overnight blood agar cultures (30°C) using the QIAGEN tissue DNA extraction kit (QIAGEN, Hombrechtikon, Switzerland), according to the manufacturer instructions.

#### Isolation of *L. sphaericus*

Water samples were serially diluted and inoculated overnight at 30°C on blood agar plates after a 15 min heat shock at 80°C to inactivate non spore-forming bacteria. One gram of each sludge sample was suspended in 10 ml of distilled sterile water, incubated for 10 min at room temperature and mixed several times using a Vortex mixer (Vortex-Genie 2, Scientific Industries, New York, USA) [37]. Serial dilutions of the sludge suspensions were then incubated as described for the water samples. Colonies with a *Lysinibacillus sphaericus* morphology [38] were further processed for a rapid identification using matrix-assisted laser desorption ionization-time of flight mass spectrometry (MALDI-ToF MS) in a MALDI-ToF Axima Confidence™ spectrometer (Shimadzu-Biotech Corp., Kyoto, Japan), as described by Benagli et al. [39], using 1 µl of α-cyano-4-hydroxycinnamic acid (CHCA; 30–40 mg ml^−1^ in 33% acetonitrile, 33% ethanol and 3% trifluoroacetic acid) matrix solution. Spectra were analyzed using SARAMIS™ (Spectral Archive And Microbial Identification System, AnagnosTec GmbH, Potsdam, Germany).

#### PFGE

Isolates were analyzed by pulsed-field gel electrophoresis (PFGE) to assess clonality. Macrorestriction profiles of isolates were compared to those of BML 2362 and *L. sphaericus* ATCC14577^T^. Bacterial cells were grown in 10 ml Tryptone Soy Broth (TSB) (Oxoid, Pratteln, Switzerland) for 24 h at 37°C with agitation. Five µl of cell suspension were re-inoculated into 5 ml of fresh TSB and incubated at 37°C until OD_590 nm_ of 1.0. Cells were harvested by centrifugation at 6500× *g* for 10 min and re-suspended in 5 ml sodium-chloride-EDTA (SE) buffer (75 mM NaCl, 25 mM EDTA). 0.4 ml of the bacterial suspension was mixed with 0.4 ml of 1.4% agarose (Pulsed Field Certified Agarose, Bio-Rad, Reinach, Switzerland) at 55°C and 100 µl aliquots were transferred to the slots of a plug mould (Bio-Rad). The plugs were incubated overnight at 37°C in 5 ml of lysis buffer (100 mM EDTA, 2 mg ml^−1^ sodium deoxycholate, 1% N-lauroylsarcosine sodium salt) containing 2 mg ml^−1^ lysozyme (Sigma, Buchs, Switzerland) and 3 U ml^−1^ mutanolysin (Sigma). The plugs were washed once with nanopure water and incubated overnight at 55°C in 5 ml of proteinase K buffer (10 mM Tris-HCL, 0.5 M EDTA, pH 8.0, 1% SDS) with addition of 2 mg ml^−1^ proteinase K (Sigma) immediately prior to use. The plugs were washed twice in nanopure water at 50°C for 30 min, and 4 times in TE buffer at 50°C for 1 h. For restriction endonuclease digestion, agarose plugs were cut to 1/3, washed in 500 µl of nanopure water for 15 min at room temperature and equilibrated in 300 µl of 1× restriction buffer A (10× SuRE/Cut Incubation Buffer A; Roche, Rotkreuz, Switzerland) at room temperature for 30 min. The restriction digest was carried out in 300 µl fresh restriction buffer A containing 50 U of restriction enzyme *Sma*I (Roche) for 20 h at 25°C. Restriction fragments were separated in a 1% Pulsed-Field Certified agarose gel (Bio-Rad) in 0.5 Tris-borate-EDTA (TBE) buffer containing 50 µM Thiourea (Sigma), using CHEF-DR III system (Bio-Rad Laboratories). The following conditions were used for the separation of the digested fragments: pulse time: 3 to 35.4 sec., linear ramping, 6 V cm^−1^, 22 h at 14°C, 120° included angle. DNA of *Salmonella* serotype Braenderup H9812 restricted during 20 h at 37°C with 40 U of *Xba*I enzyme in 300 µl of 1× restriction buffer B (10× SuRE/Cut Incubation Buffer B; Roche, Switzerland) was used as size marker. Following electrophoresis, the agarose gel was stained in ethidium bromide solution (5 mg l^−1^ in distilled water) for 30 min and destained in distilled water for 30 min. Fingerprint patterns were visualized and captured under UV light using a CCD photography system (Gel Doc 2000 system, Bio-Rad, Hercules, CA).

#### Sequencing of 16S rRNA gene

The partial sequence of the 16S rRNA gene was analyzed by amplifying a 1.4 kb DNA fragment using the universal primers uniL 26f (forward primer, nucleotide sequence 5′ - ATTCTAGAGTTTGATCATGGCTCA - 3′) and uniR 1392r (reverse primer, nucleotide sequence 5′ – ATGGTACCGTGTGACGGGCGGTGTGTA - 3′) [40]. Amplifications of the 16S rRNA gene were performed in a final volume of 50 µl containing 5 µl of 10× PCR buffer (QIAGEN), 1.5 mM MgCl_2_, 1 µl PCR Nucleotide Mix Plus (Roche), 0.2 µM of each primer, 1.25 U Taq polymerase (250 U HotStarTaq DNA Polymerase, QIAGEN) and 3 µl of template DNA. Reactions were performed in a Veriti® 96-well Thermal Cycler (Applied Biosystems). The PCR conditions used were 15 min at 94°C, 35 cycles of 30 s at 94°C, 1 min at 52°C and 90 s at 72°C, followed by a final extension at 72°C for 7 min. The PCR products were purified using 900 µl Sephadex® G-100 (Sigma) columns by centrifugation at 770× *g* for 3 min. The sequence reactions were performed in both directions using the BigDye® Terminator v3.1 Cycle Sequencing Kit (Applied Biosystems). The reactions consisted of a final volume of 10 µl containing 1 µl BigDye® Terminator Ready Reaction Mix, 1.5 µl of 5× BigDye® Buffer, 0.2 µM of either uniL 26f or uniR 1392r primer, and 30 ng DNA. The temperature profiles were as for 16S rRNA gene amplification. Sequencing products were subsequently purified using 900 µl Sephadex® G-50 (GE Healthcare Life Sciences, Glattbrugg, Switzerland) columns. Ten µl of purified sequencing product were mixed with 5 µl of HiDi Formamide (Applied Biosystems) and resolved on an AB 3500 Genetic Analyzer (Applied Biosystems). 16S rRNA gene sequences were analyzed using MEGA version 4 [41]. Sequences were submitted to GenBank (National Center for Biotechnology Information, Maryland, USA) and assigned the accession numbers JX535354, JX535355, JX535356, JX535357, and JX535358. Sequences from this study were aligned with other reference gene sequences retrieved from the GenBank database using ClustalW [42]. A phylogenetic tree was constructed on the basis of the neighbour-joining algorithm with bootstrap analysis based on 1,000 replications.

#### *BinA* gene

The presence of the *binA* gene was analyzed by real-time PCR as described above. Strains 2362 and the ATCC14577^T^ were used as positive and negative controls, respectively.

### Data analysis

The 95% LOD for both real-time PCR targeting the Bti *cry4* genes and the Ls *binA* gene were determined using a Probit regression analysis. The concentrations of Bti and Lsph spores per gram of sludge or ml water were log_10_(x+1) transformed. If not stated otherwise, data are presented as average±standard error (SE). Positive samples with concentrations above the LOD but below the LOQ were excluded from the analysis. All samples below the LOD were considered negative. Data obtained from samples collected in catch basins with high concentrations of Bti and Lsph spores prior to treatment were removed from the analysis. One-way analysis of variance (ANOVA, α set to 0.05) with Tukey's HSD post-hoc correction were carried out to test for significance of the differences between Bti and Lsph concentrations over time. All statistical analyses were carried out using SPSS ver. 17.0.1 for Windows (SPSS Inc., Chicago, IL, USA).

## Results

### Quantitative real-time PCR performances

The analytical sensitivity of the real-time PCR assay was high for both *Bacillus thuringiensis israelensis* and *Lysinibacillus sphaericus*. The 95% LOD for the Bti assay was 2.04 spores per reaction, corresponding to 2.0·10^3^ Bti spores per gram of sludge and 1.3·10^3^ spores per ml of water. The 95% LOD for Lsph was slightly higher, with 5.95 spores per reaction, equivalent to 6.0·10^3^ spores per gram of sludge and 3.7·10^3^ spores per ml of water.

**Figure 1:**
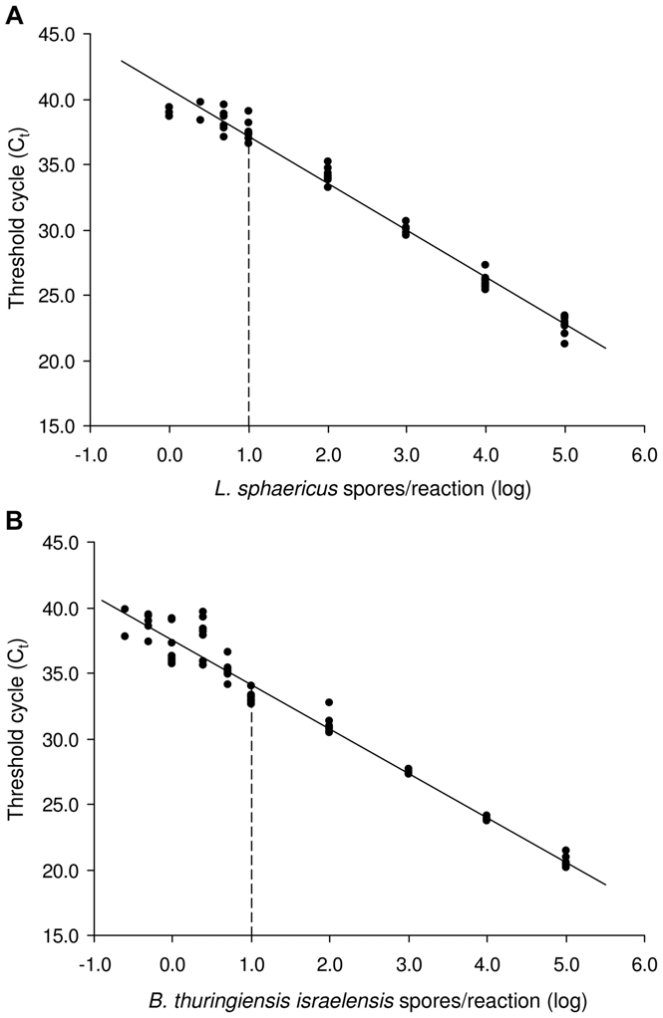
Standard curves for the quantification of *Lysinibacillus sphaericus* (A) and *Bacillus thuringiensis israelensis* (B). Each spore concentration was tested with 9 replicates on 3 different runs. Linear regressions, Lsph: y = 3.59x+40.71, R^2^ = 0.98; Bti: y = 3.39x+37.50, R^2^ = 0.97. The dashed line represents the LOQ of the real-time PCR assay.

The accuracy of the quantitative real-time PCR assays was determined according to the RSD among standard replicates. The lower the standard DNA concentration, the higher was the variation within replicates (Figure 1). The LOQ (i.e. the lowest concentration tested with a RSD below 25%) was set to 10 spores per reaction for both real-time PCR systems, equivalent to 6.3·10^3^ spores per ml of water and 1.0^.^10^4^ spores per gram of sludge.

Filtration of water samples prior to DNA extraction allowed the sensitivity of the method to be increased. The number of spores that could be detected and reliably quantified in the original samples was in the order of 10 spores per ml.

### Analysis of water and sludge samples

#### Water

**Figure 2:**
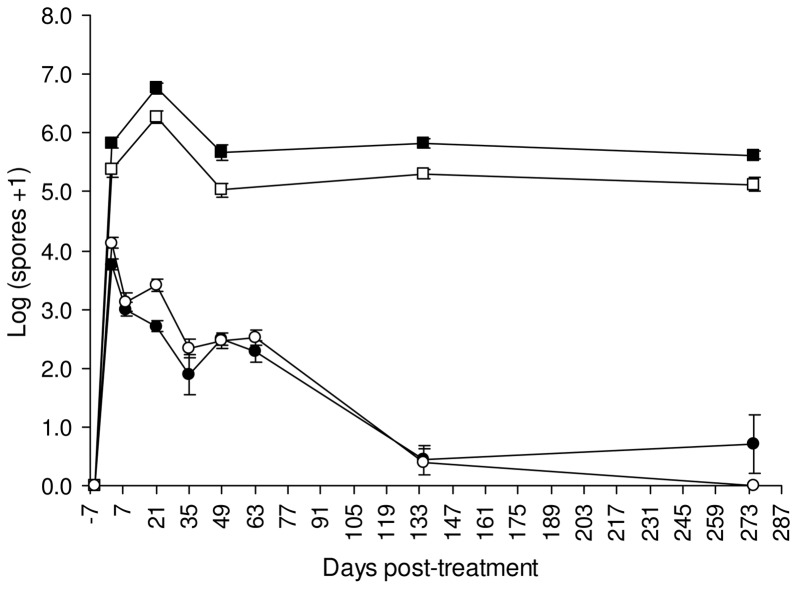
Evolution of *Lysinibacillus sphaericus* and *Bacillus thuringiensis israelensis* spores in water and sludge samples collected in treated catch basins (average±SE). Symbols: ○ = Lsph spores per ml of water; • = Bti spores per ml of water; □ = Lsph spores per gram of sludge; ▪ = Bti spores per gram of sludge.

The number of Bti and Lsph spores in water increased significantly after BML application to catch basins, to decline progressively (Figure 2). Five days prior to treatment, no Bti spores were detected in water, while Lsph spores were discovered in two catch basins (T9 and T11), with concentrations of 2.37±0.02 log and 3.59±0.02 log per ml of water, respectively. These catch basins were accordingly not considered in the analysis of the dynamic of spores. Two days after the treatment, the number of spores attained maximal levels, with 3.76±0.08 log Bti and 4.13±0.09 log Lsph per ml. From day 2 post-treatment, the concentration of spores declined progressively, reaching values similar to pre-treatment samples at day 135 post-treatment for both Lsph (0.40±0.22 log per ml; Tukey's HSD test, p = 0.50) and Bti (0.44±0.24 log per ml; Tukey's HSD test, p = 0.51). At day 135 post-treatment, the concentration of Lsph spores quantified in catch basin T11 were similar to that before the treatment, with 3.54±0.01 log per ml of water. At the end of the experiment, no Lsph spores were detected in the water of the four treated catch basins sampled.

For water samples, some data were removed from the analysis because the number of spores detected by real-time PCR was below the LOQ (see Figure S1 in Supporting Information).

#### Sludge

Five days before BML treatment, high concentrations of Bti spores were measured in sludge samples from catch basins T8 (4.50±0.07 log per gram) and T10 (4.11±0.04 log per gram). The same was true for Lsph spores, with 5.03±0.01 log per gram of sludge from catch basin T11. These catch basins were accordingly not considered in the analysis of the dynamic of spores. Lsph was detected also in catch basin T4, but with concentrations below the LOQ. After BML application, the concentration of Bti and Lsph spores in sludge increased, remaining significantly higher until the last sampling (Tukey's HSD test, p<0.05) (Figure 2). At day 2 post-treatment, the number of Bti and Lsph spores was 5.83±0.01 log and 5.36±0.12 log per gram of sludge, respectively. A slight, but significant (Tukey's HSD test, p<0.05) increase was observed 21 days after the treatment, with 6.75±0.10 log Bti spores and 6.27±0.11 log Lsph spores per gram of sludge. At day 275 post-treatment, the concentration of both Bti and Lsph spores did not differ from that measured 2 days after the treatment (Tukey's HSD test, p = 0.88 and p = 0.80 for Bti and Lsph, respectively). All sludge samples collected after BML treatment gave positive amplification signals with real-time PCR, with concentrations above the LOQ.

Concentrations of Bti spores above the LOQ were measured in sludge samples of the untreated control catch basins UTC1 and UTC2, with an average of 4.59±0.39 log per gram along the test period. All UTC samples were negative for Lsph.

### Isolation and characterization of *L. sphaericus*

Spore-forming bacteria were isolated from water and sludge samples of catch basins T4, T9 and T11 collected before BML treatment. 11 isolates were identified as *Lysinibacillus sphaericus* using MALDI-TOF MS. Of them, 2 were recovered from sludge of catch basin T4 (isolates 4.1 and 4.2), 3 from water samples of catch basins T9 (isolates 9.1, 9.2, and 9.3), and 6 from catch basin T11 (3 from water: 11.1, 11.2, 11.3; and 3 from sludge: 11.4, 11.5, and 11.6). All isolates from catch basins T9 and T11, as well as isolate 4.1 from catch basin T4, were identified as *L. sphaericus* using the SARAMIS™ database.

**Figure 3:**
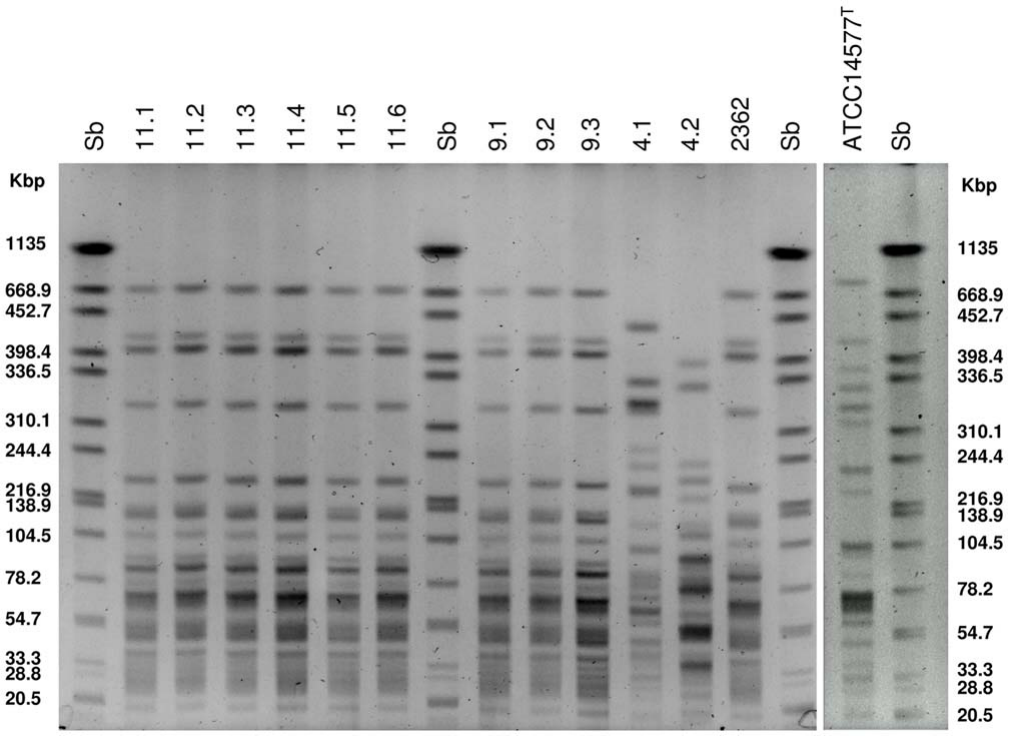
PFGE patterns of *Sma*I digested genomic DNA of the 11 *Lysinibacillus* isolates and references (strains 2362 and the ATCC14577^T^). Sb: *Salmonella* serotype Braenderup H9812 restricted with *Xba*I.

Pulsed field gel electrophoresis analysis performed with the isolates from catch basins, as well as with strains 2362 and ATCC14577^T^, generated 4 *Sma*I patterns (Figure 3). The reference ATCC14577^T^ produced a unique profile. Among the other isolates, three different *Sma*I profiles were obtained (Figure 3). Both strains 4.1 and 4.2 were unrelated to all the isolates tested, as well as to strain 2362 and ATCC14577^T^, yielding profiles that differed by more than 7 bands. All the isolates from catch basins T9 and T11, as well as strain 2362 were indistinguishable, having an identical *Sma*I profile (Figure 3).

Enterobacterial Repetitive Intergenic Consensus (ERIC) PCR was also performed with the 11 isolates and the reference strain 2362. Four distinct profiles were obtained, with a unique pattern retrieved for isolates from catch basins T9, T11, as well as for strain 2362, confirming the results obtained by PFGE analysis (data not shown).

**Figure 4:**
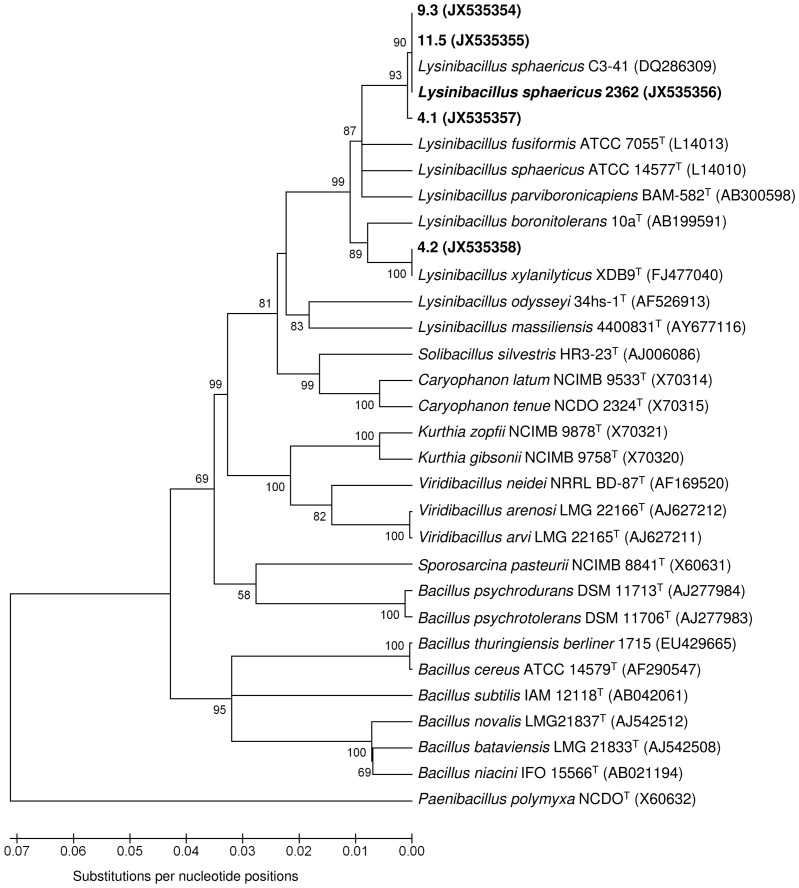
Neighbour-joining phylogenetic tree based on 16S rRNA gene sequences of isolates 4.1, 4.2, 9.3 and 11.5, as well as strain 2362 and some other strains belonging to related taxa. The evolutionary distances are based on the Kimura 2-parameter model. Bootstrap values (>50%) based on 1000 replications are shown at branch nodes. Gene Bank accession numbers are given in parentheses. *Paenibacillus polymyxa* NCDO 1774^T^ was used as outgroup. Phylogenetic analysis was performed using MEGA version 4.

The analysis of the *binA* gene and the sequencing of the 16S rRNA gene were carried out with one representative isolate for each different PFGE profile. Strain 2362 was used for comparison. Isolates 9.3 and 11.5 gave positive amplifications for the *binA* toxin gene, whereas no PCR signals were obtained for isolates 4.1 and 4.2. Analysis of the 16S rRNA gene confirmed that isolates 9.3 and 11.5 are *L. sphaericus*, with 100% of sequence homology with the highly pathogenic strains 2362 and C3-41 used for mosquito control (Figure 4). Isolate 4.1 was also confirmed to belong to the species *L. sphaericus*, with only 2 nucleotide substitutions in the amplified 16S rRNA gene sequence compared to strain 2362. 16S rRNA gene analysis revealed that isolate 4.2 belongs to the species *Lysinibacillus xylanilyticus*, with 100% sequence homology to the type strain XDB9 (sequence retrieved from GenBank) (Figure 4).

## Discussion

This is the first report of the use of a real-time PCR-based methodology for the quantification of *L. sphaericus* spores in environmental samples. In the present study we evaluated the dynamics and persistence of *Bacillus thuringiensis* var. *israelensis* and *Lysinibacillus sphaericus* spores applied to urban catch basins. For this purpose, a sensitive and quantitative real-time PCR for the detection and quantification of *L. sphaericus* spores was designed, adapting methods developed in previous studies for Bti [30], [32]. Previous evaluations of the persistence and settling of Lsph after application to water columns or mosquito breeding sites relied on classical plating procedures for spore counts [24], [26], [43]. Molecular methods based on DNA extraction directly from the environmental samples followed by real-time PCR quantifications of the target organism have the advantage to provide high specificity, sensitivity and speed, making them suitable for the monitoring of microbial biocontrol agents in the environment. The precision and analytical sensitivity of the method was similar for both Bti and Lsph spores, with limits of detection (95% LOD) and quantification (LOQ) for sludge samples in the order of 10^3^ and 10^4^ spores per gram, respectively. Filtration of water samples prior to DNA extraction allowed the sensitivity of the PCR to be increased, with LOQ and LOD in the order of 10 spores per ml water. The real-time PCR used provided sensitive and fast quantification of both Bti and Lsph, and could be applied to monitor the concentration of spores in water and sludge of catch basins before and after BML treatment, over a period of at least 280 days.

With this method, we could show that the concentration of Bti and Lsph in water increased after BML application, to decrease gradually to pre-treatment levels. This could be due to the progressive sedimentation of the spores. Two days after treatment, most Bti and Lsph spores were found in the bottom sludge where they persisted at high levels, with spore counts remaining rather constant for at least 275 days. In accordance with our findings, Davidson and collaborators [24] reported a rapid sedimentation of *L. sphaericus* spores applied to field plots and accumulating in the bottom sludge. *L. sphaericus* 2362 spores persisted for more than 5 months in the bottom mud of cesspools, with concentrations of up to 8·10^3^ spores per ml [26]. An increase in the number of both Bti and Lsph spores was observed 3 weeks after BML treatment, excluding a simple persistence of the introduced spores without growth. However, 275 days after treatment, the number of spores reached numbers close to those measured at day 2 post-treatment, suggesting that dilution through rainfalls, predation, and recycling through spore germination and vegetative growth have no major influence in reducing or increasing their number on the long period.

Bti and Lsph were detected in some untreated control samples collected before BML application. Treatments with a biolarvicide containing only Bti were carried out in the past for the control of *Aedes albopictus*. This could explain the presence of Bti in sludge samples from two catch basins prior to BML application. On the other hand, products based on the entomopathogenic *L. sphaericus* have never been used before in the settings studied here. Accordingly, *L. sphaericus* strains detected and isolated from this area are indigenous. *L. sphaericus* is an ubiquitous bacterium, found in several soil and aquatic habitats [3]. PFGE and ERIC PCR analysis of genomic DNA, as well as 16S rRNA gene sequence analysis, have shown that nine isolates, obtained from two close catch basins located within the same city block, are genetically indistinguishable from strain 2362, indicating that they are indigenous isolates belonging to the same clonal population of strain 2362. PFGE of chromosomal DNA digested with appropriate restriction enzymes represent an accurate and reproducible typing technique for the identification of closely related bacterial isolates [44]. Strains with restriction profiles differing by less than three bands are considered to be closely related and arising from the same ancestor and members of the same clone [44]. The original clonal structure of *L. sphaericus* was previously demonstrated by PFGE [45] and by multilocus enzyme electrophoresis (MLEE) sequence typing [46]. A recent multi-locus sequence typing (MLST) study confirmed that there is a clonal population structure in the *L. sphaericus* toxic strains, while non-toxic strains are considerably more heterogeneous [47]. Serotype 5a5b is the most frequent among all entomopathogens of group IIA and represents a widely disseminated clone, with strains isolated from all parts of the world [48]. *L. sphaericus* strains with serotype 5a5b were associated with the same PFGE *Sma*I profile [45] and include the highly pathogenic strains commercialized for mosquito control, such as 2362 and C3-41 [6].

Two isolates from a single catch basin were found to be indigenous strains unrelated to strain 2362. Isolate 4.1 was found to be a *L. sphaericus* strain. The absence of amplification for the *binA* gene in this strain could be attributed to genetic polymorphism which prevented the annealing of the primers used. On the other hand, it could also be an indigenous *L. sphaericus* lacking the *binA* gene, indicating that this strain might be either non-pathogenic or possesses a low toxicity toward mosquito larvae. The phylogenetic tree based on the 16S rRNA gene sequences showed that isolate 4.1 is more related to strains 2362 and C3-41 than to *L. sphaericus sensu stricto* (ATCC 14577) and *L. fusiformis* (ATCC 7055) type strains, suggesting that it could belong to subgroup IIA. Non-pathogenic strains of group IIA lacking *bin* and *mtx1* toxin genes are more common in the environment than toxic types [49]. Isolate 4.2 was improperly identified as *L. sphaericus* using the MALDI-TOF MS SARAMIS™ database. Further analysis of the 16S rRNA gene sequence led to the classification of this isolate as *Lysinibacillus xylanilyticus*, a species recently proposed by Lee et al. [50].

In conclusion, a specific and sensitive real-time PCR was developed for the detection and quantification of *L. sphaericus* spores in environmental samples. We used this method to evaluate the dynamics of *B. thuringiensis* var. *israelensis* and *L. sphaericus* spores in water and sludge samples following a BML application to urban catch basins. The absence of a significant increase in the number of spores as a result of recycling underlines the environmental safety of this microbial biopesticide.

## Supporting Information

Figure S1 **Percentage of water samples positive for** ***Lysinibacillus sphaericus*** **(A) and** ***Bacillus thuringiensis israelensis*** **(B).** Black: percentage of positive samples with concentrations above LOQ; grey: positive samples with concentrations below LOQ that were removed from the statistical analysis.
(TIF)
