# Supplementary figures and images for: Dynamics of Bacillus thuringiensis var. israelensis and Lysinibacillus sphaericus Spores in Urban Catch Basins after Simultaneous Application against Mosquito Larvae
Source: PLoS One. 2013 Feb 4;8(2):e55658. doi: 10.1371/journal.pone.0055658 (PMC3563526; doi:10.1371/journal.pone.0055658)

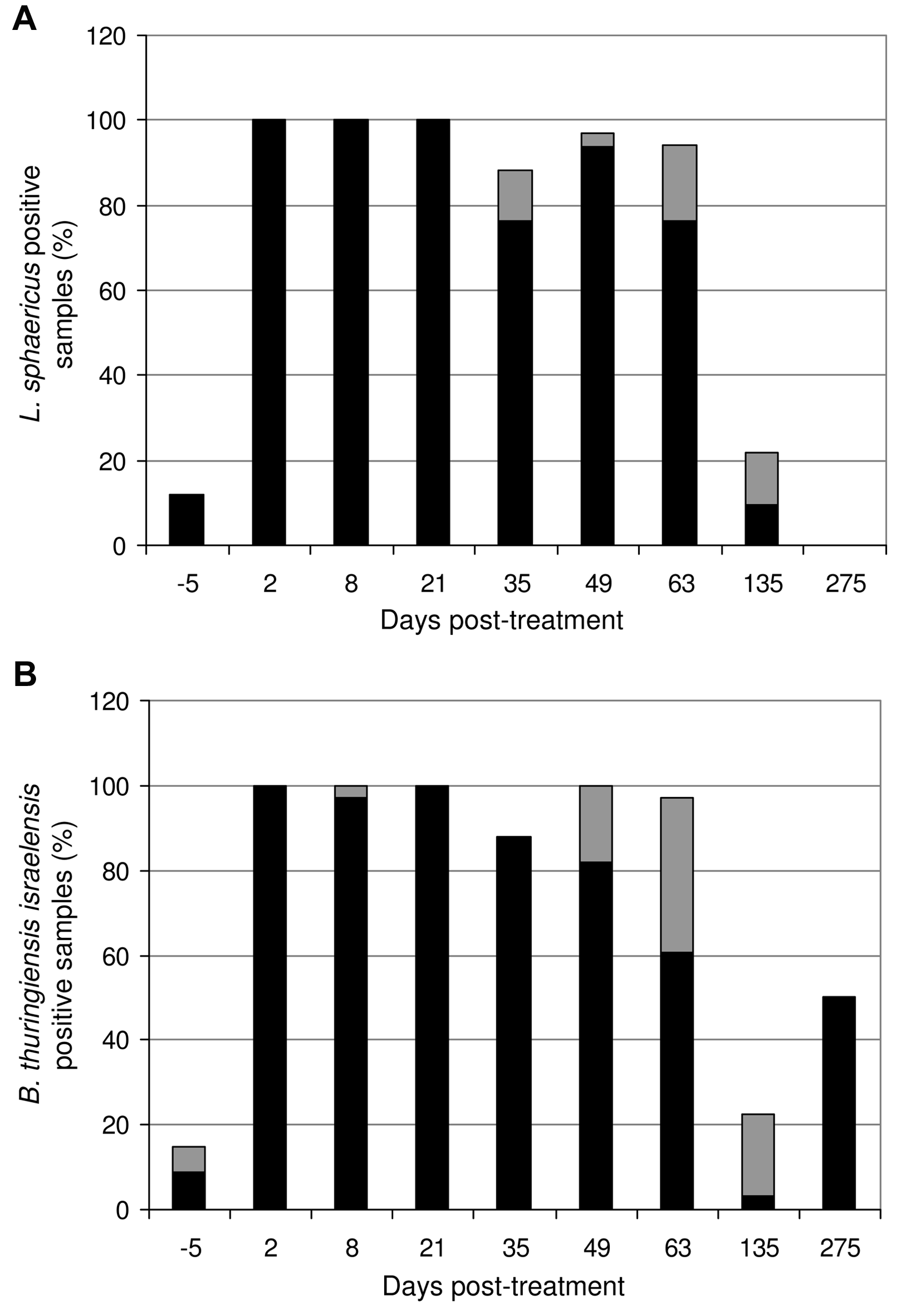

Supplement: Figure S1 — Percentage of water samples positive for Lysinibacillus sphaericus (A) and Bacillus thuringiensis israelensis (B). Black: percentage of positive samples with concentrations above LOQ; grey: positive samples with concentrations below LOQ that were removed from the statistical analysis. (TIF) [file pone.0055658.s001.tif]
